# Thrombolytic Enzymes of Microbial Origin: A Review

**DOI:** 10.3390/ijms221910468

**Published:** 2021-09-28

**Authors:** Deepti Diwan, Zeba Usmani, Minaxi Sharma, James W. Nelson, Vijay Kumar Thakur, Graham Christie, Gustavo Molina, Vijai Kumar Gupta

**Affiliations:** 1 Department of Neurosurgery, Washington University School of Medicine, Saint Louis, MO 63110, USA; diwand@wustl.edu (D.D.); jwnelson@wustl.edu (J.W.N.); 2 Department of Applied Biology, University of Science & Technology, Techno City, Killing Road, Baridua 9th Mile 793101, Meghalaya, India; zeba24@gmail.com (Z.U.); minaxi86sharma@gmail.com (M.S.); 3 Biorefining and Advanced Materials Research Center, SRUC, Edinburgh EH9 3JG, UK; vijay.thakur@sruc.ac.uk; 4 School of Engineering, University of Petroleum & Energy Studies (UPES), Dehradun 248007, Uttarakhand, India; 5 Department of Chemical Engineering & Biotechnology, University of Cambridge, Cambridge CB2 1TN, UK; gc301@cam.ac.uk; 6 Laboratory of Bioflavors and Bioactive Compounds, Department of Food Science, Faculty of Food Engineering, State University of Campinas, R. Monteiro Lobato, 80, Campinas, São Paulo 13083-862, Brazil; gustavomolinagm@gmail.com; 7 Centre for Safe and Improved Food, SRUC, Edinburgh EH9 3JG, UK

**Keywords:** fibrinogen, fibrin, thrombolytic therapy, microbial enzymes, thrombosis, hemostasis

## Abstract

Enzyme therapies are attracting significant attention as thrombolytic drugs during the current scenario owing to their great affinity, specificity, catalytic activity, and stability. Among various sources, the application of microbial-derived thrombolytic and fibrinolytic enzymes to prevent and treat vascular occlusion is promising due to their advantageous cost–benefit ratio and large-scale production. Thrombotic complications such as stroke, myocardial infarction, pulmonary embolism, deep venous thrombosis, and peripheral occlusive diseases resulting from blood vessel blockage are the major cause of poor prognosis and mortality. Given the ability of microbial thrombolytic enzymes to dissolve blood clots and prevent any adverse effects, their use as a potential thrombolytic therapy has attracted great interest. A better understanding of the hemostasis and fibrinolytic system may aid in improving the efficacy and safety of this treatment approach over classical thrombolytic agents. Here, we concisely discuss the physiological mechanism of thrombus formation, thrombo-, and fibrinolysis, thrombolytic and fibrinolytic agents isolated from bacteria, fungi, and algae along with their mode of action and the potential application of microbial enzymes in thrombosis therapy.

## 1. Introduction

**Figure 1:**
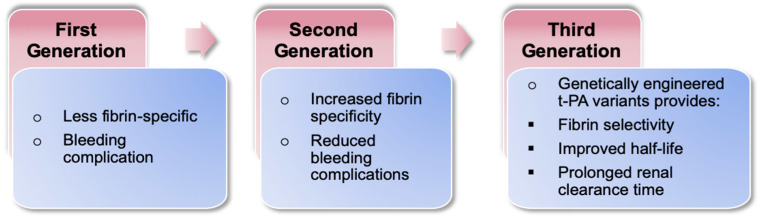
Generational classification and features of thrombolytic drugs.

Dysregulation of the intrinsic balance between the coagulation cascade and the fibrin- and thrombolytic pathways can lead to severe outcomes such as myocardial infarction, stroke, and massive pulmonary embolism caused by fibrin accumulation in the blood vessel walls, resulting in the formation of hemostatic plug or a clot [1]. Currently, the tissue plasminogen activator (tPA) or plasminogen activator (PA) is an indispensable clinical tool in thrombolytic therapy [2]. They are categorized into first, second, and third generation (Figure 1) [3]. PA are serine proteases that form active plasmin from inactive plasminogen and mediate fibrin lysis through two modes of action: (i) direct—channels direct action on plasminogen to catalyze its activation; and (ii) indirect—forms a 1:1 stoichiometric complex with plasminogen or plasmin followed by activation of the circulating plasminogen molecule. Despite their clinical effectiveness, however, these agents have several undesirable side effects, including immunogenicity, gastrointestinal bleeding, intracranial hemorrhage, and major systemic hemorrhage [4,5]. Consequently, there is an urgent need for approaches exploring new fibrinolytic agents with better specificity and efficacy.

There are several known sources of fibrinolytic enzymes, including plants, animals, and microbes. Among them, microbial-derived fibrinolytic enzymes have gained particular interest because they possess the following advantages: (i) cost-effective production, (ii) fewer to no side effects [6], (iii) broad biochemical diversity, (iv) mass-culture feasibility, and (v) allow genetic manipulation. Hence, various microorganisms have been used for the isolation of fibrinolytic enzymes, including bacteria, fungi, and algae.

The purpose of this review is to provide an overview of the microbial-derived enzymes with thrombolytic and fibrinolytic potential and summarize the existing literature on the microbes proposed to produce enzymes with thrombolytic and fibrinolytic activities.

### 1.1. Mechanism of Thrombus Formation

Hemostasis is defined as the physiological process of cessation of blood loss by clot formation at the site of an injury. Fibrin is a major component of blood clots, and is formed from fibrinogen (large soluble plasma glycoprotein) via proteolysis by thrombin. The accumulation of fibrin clots results in thrombus formation. A thrombus is a blood clot formed within the blood vessel [7]. Under physiological conditions, a proteolytic enzyme—plasmin—hydrolyses the fibrin clot to prevent thrombosis in blood vessels. During vascular pathophysiology or damage to the vascular system, the process of clot hydrolysis is disrupted, which may result in thrombosis [8].

**Figure 2:**
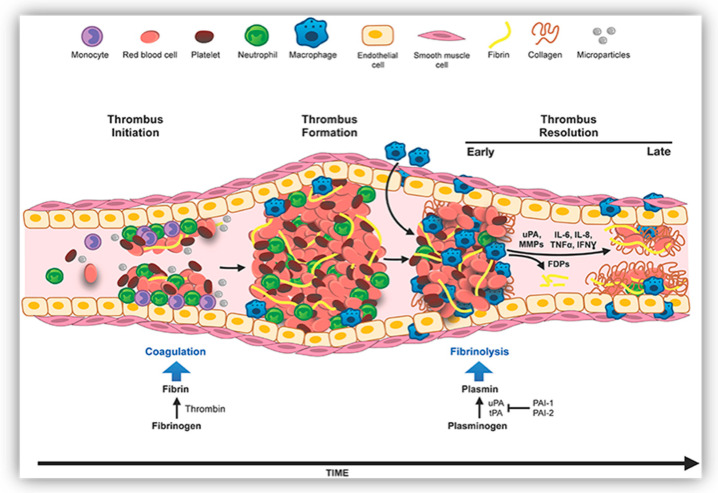
Innate immune cells in deep vein thrombosis (DVT). Adapted with permission from Mukhopadhyay et al., copyright © 2019 [11].

Hemostasis and thrombosis are intricate, multifactorial processes. Platelets, in conjugation with endothelial cells and coagulation proteins, are the crucial mediator of vascular hemostasis and thrombosis. Disruption to any of these processes could result in atherosclerotic plaque formation [9,10,11] (Figure 2, leading to variety of thrombotic diseases including CVDs (cardiovascular diseases) [12,13], abdominal aortic aneurysms (AAAs) [14,15], pulmonary embolism (PE) [16], and stroke [17,18].

The process of hemostasis is divided into two stages: (1) primary (involves rapid platelet activation) and (2) secondary (requires additional coagulation pathways to form polymeric fibrin) processes (Table 1).

### 1.2. Fibrinolysis and Thrombolysis

Fibrinolysis is the breakdown of fibrin in the blood clot, and thrombolysis can be simply defined as the process of thrombus dissolution.

Fibrin is a primary protein component of a blood clot that is formed from fibrinogen (340 kDa glycoprotein) by thrombin-mediated proteolysis and elimination of N-terminal fibrinopeptides from the Aα and Bβ chains. A serine protease, plasmin, is an activated form of inert plasma precursor, plasminogen (PLG). PLG is a 791-AA-long glycoprotein that circulates in blood plasma as an inactive zymogen. The plasmin is a crucial enzyme in the dissolution of fibrin clots. There exist two major glycoforms of human plasminogen, namely, type I (consists of two molecules of glycosylation) and type II (consists of one molecule of glycosylation) [8,22]. The binding of circulating PLG to the cell surface or blood clot acquires an open conformation, which aids in the formation of catalytically active plasmin by the Arg561-Val562 bond cleavage via a direct PLG activator [23]. Direct PA includes tissue plasminogen activator (tPA), streptokinase and urokinase or their variants [3,24]. Plasmin formation leads to fibrin lysis, giving soluble fibrin degradation products (FDPs) [22,25]. Therefore, the process of fibrinolysis involves two phases: (i) PLG activation on the surface of the fibrin clot to forms plasmin, which dissolves fibrin—i.e., a physiological process; and (ii) amplification of plasmin-induced clot breakdown by exposing the additional binding sites of degraded fibrin—i.e., pharmacologically mediated processes such as fibrinolytic and thrombolytics.

## 2. Microbes in Thrombolytic Therapy

Many microbial-derived thrombolytic enzymes have been discovered and characterized. In general, the strains that produce thrombolytic enzymes are screened and isolated by using sterile skimmed milk agar with subsequent incubation of culture at 37 °C for 24 h; protease-positive isolates form a hydrolytic zone, which is screened by fibrin plate assay [26,27,28,29]. The fibrin plate assay is a precise and sensitive method to quantitate the extent of fibrin breakdown. Briefly, a fibrin clot covering the bottom of a petri dish is treated with the fibrinolytically active solutions, and then the relative amounts of fibrin (converted substrates) are measured by determining the areas of the lysed zones. These enzymes are produced via solid and liquid state fermentation in a medium enriched with carbon sources (such as cellulose, dextran, dextrose, fructose, starch, maltose, ribose, sucrose, galactose, and trehalose) and nitrogen sources (including organic-based (yeast extract, peptone, gelatin, urea, casein, tryptone, and soya meal) and inorganic salts (ammonium chloride, sodium dihydrogen phosphate, ammonium sulfate, ferrous sulfate, potassium nitrate, calcium chloride, ferrous sulfate, and disodium hydrogen phosphate)). Inhibitors such as ethylene glycol-bis(β-aminoethyl ether)-N,N,N′,N′-tetraacetic acid (EGTA), phenylmethylsulfonyl fluoride (PMSF), and ethylenediaminetetraacetic acid (EDTA); and metal ions (Cu^2+^, Co^2+^, Ca^2+^, Fe^3+^, Fe^2+^, K^+^, Mg^2+^, Mn^2+^, Na^+^, and Zn^2+^) are supplemented in the culture medium to further enhance the production process of thrombolytic enzymes [30,31]. One of the physiological advantages of microbial-derived thrombolytic enzymes is their stability at a wide range of pH values (5–11) and temperatures (30–70 °C). In addition, molecular cloning approaches abet the characterization of genes encoding target-specific modes of action with minimal side-effects, facilitating the potent application of thrombolytic enzymes in warfare for blood clotting disorders, discussed elsewhere in greater detail [32,33].

To overcome the associated drawbacks of currently available thrombolytic agents, various preliminary evaluation approaches are used to test the pharmacokinetics and dynamics of potential drug candidates of microbial origin with respect to their pharmacological cogency and clinical relevance. Briefly, in vitro and in vivo assays to assess the clot lysis properties of these agents are performed either by using fibrin as a direct substrate or by the production of a fibrin clot using thrombin.

The in vitro assays include: (i) *Antiaggregatory activity*—measures reduction or inhibition of platelet aggregation [34]; (ii) *Anticoagulant and thrombolytic activity*—measures ability to prevent blood clot formation and dissolution of an existing blood clot [35,36]; (iii) *Clot formation and lysis assay (CloFAL)*—periodic quantitative measurement of clot lysis, aid in the clinical evaluation of net hemostatic balance, and bleeding and clotting disorders [37]; (iv) *Euglobulin clot lysis assay*—rapid and sensitive method of measuring fibrinolysis within the euglobulin fraction [38]; and (v) *Thromboelastography (TEG)*—the ability to measure the viscoelastic properties of the whole blood clot [39].

The in vivo assays include: (i) *Carrageenan-induced thrombosis model*—helps assessment of clinically relevant anti-thrombus and thrombolytic agents by demonstrating disappearance of wine-colored thrombus [40]; (ii) *D*—*Dimer test*—fibrin derivatives containing cross-linked D—dimer (XDP) domains help monitor fibrinolysis [41]; (iii) *Ferric chloride-induced thrombosis model*—aids in assessing anti-platelet and anticoagulant drugs [42]; and (iv) *Rat groin flap model*—an important tool in comparative analysis of various anticoagulants and vasomotor drugs [43]. In addition to promising a better understanding of the role of the drug candidate in alleviating disease pathophysiology and determining its therapeutic potential, the combination of in vitro and in vivo assays also provides propitious translational application of microbial-derived novel thrombolytic agents.

### 2.1. Promising Microbial Producers of Thrombolytic Enzymes

The past decade has witnessed a surge in the discovery of thrombolytic agents from numerous microbes, and this has kindled the development and characterization of microbial-derived agents with thrombolytic activity and minimal or no side effects. The known microbial producers of thrombolytic enzymes include bacteria, fungi, and algae.

#### 2.1.1. Bacteria

**Figure 3:**
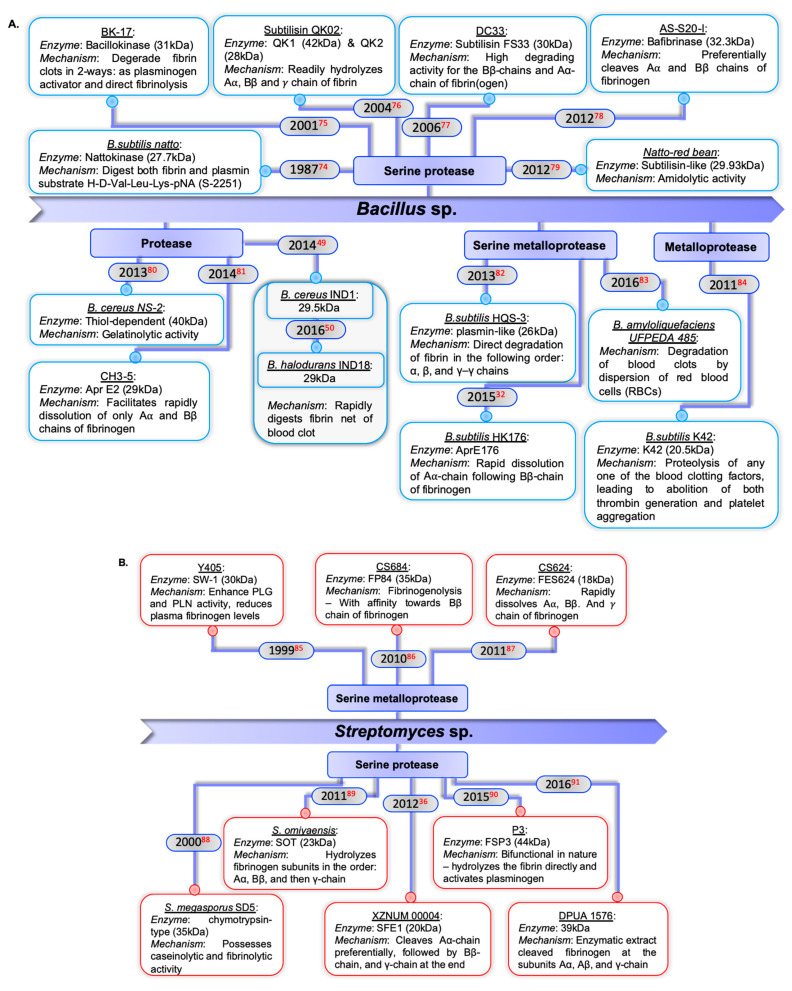

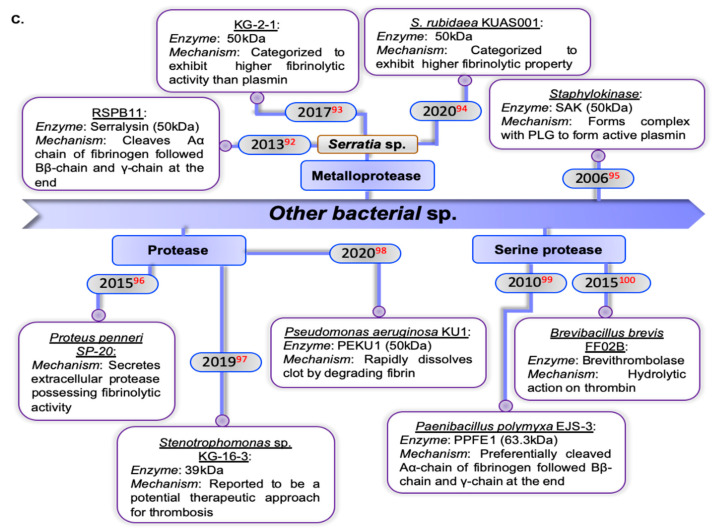
Bacterial-derived fibrinolytic enzymes and their mode of action. (**A**) *Bacillus* sp. [32,49,50,74,75,76,77,78,79,80,81,82,83,84]; (**B**) *Streptomyces* sp. [36,85,86,87,88,89,90,91]; (**C**) other bacterial species [92,93,94,95,96,97,98,99,100].

Bacteria are the first-line sources because bacterial proteins are suitable for oral administration and facilitate large-scale production. (a) *Bacillus* sp. are among the most preferred sources; various strains have been reported to have fibrinolytic activity (Figure 3A). In addition, several other strains with fibrinolytic properties have been reported, although their mode of action is yet to be elucidated, such as *Bacillus* sp. DJ–2 [44], *B. subtilis* A26-derived subtilisin BSF1 and BAF1 obtained from *B. amyloliquefaciens* An6 [45,46], enzyme URAK produced by *B. cereus* NK1 [47], *B. cereus* GD 55-derived protease [48], *B. cereus* IND1 [49] and *B. halodurans* IND18 [50] are the sources of proteolytic enzymes that exhibit both thrombolytic activity and PLG activation properties, while a fibrinolytic protease with absolute clot dissolution ability in a short span of time (within 4 h) in in vitro conditions was obtained from *Bacillus* sp. IND12 [51], *B. pseudomycoides* strain MA02 [52], and *B. cereus* RSA1 [53]; (b) *Streptomyces* sp. are the largest fibrinolytic enzyme-producing genus. Figure 3B summarizes the fibrinolytic agents derived from different strains of *Streptomyces* sp.; (c) other bacterial sp. that have been reported to produce fibrinolytic enzymes are summarized in Figure 3C. Additionally, a proteolytic enzyme of ~50 kDa serrapeptase (SP) or serralysin derived from enterobacterium *Serratia* E–15 [54] was noted to have fibrinolytic potential [55] along with the ability to distinguish and dissolve only dead and damaged tissue without harming the living tissue [55], *Treponema denticola* was used to produce thrombolytic enzymes [56], proteases of 44 kDa and 64 kDa obtained from *Shewanella* sp. IND20 and *Psuedoalteromonas* sp. IND11, respectively, showed direct clot lysis activity as well as PLG activation ability [57,58], *Paenibacillus* sp. IND8 [59], and *Stenotrophomonas maltophilia* Gd2 were shown to possess robust fibrinolytic activity [30].

#### 2.1.2. Fungi

**Figure 4:**
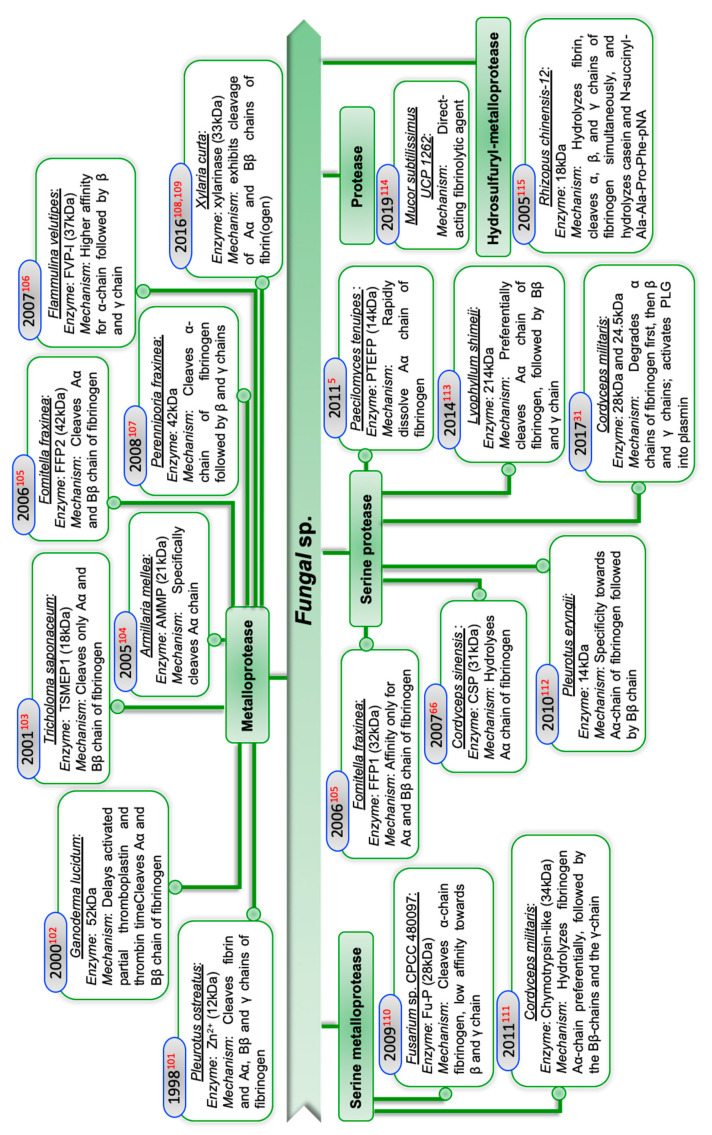
Fungal-derived enzymes with fibrinolytic potential and their mechanism of action [5,31,66,101,102,103,104,105,106,107,108,109,110,111,112,113,114,115].

The enzymatic properties of fungal sp. are a novel and largely unexplored facet, and there exists exiguous literature on the fibrinolytic potential of these microorganisms. Fungi are considered a suitable source of thrombolytic enzymes because of their ability to grow on solid substrates like agro-industrial waste residues. Different fibrinolytic agents obtained from fungal species and their mechanism of action are outlined in Figure 4. Furthermore, *Cochliobolus lunatus* and *Penicillium chrysogenum* H9 were documented as fibrinolytic enzyme producers [60,61], metalloprotease with plasmin-like activity was produced by *Fusarium pallidoroseum* [62], purified *Aspergillus ochraceus* 513 was reported to possess both fibrinolytic and anticoagulant potential [63], *Oidiodendron flavum* is another fibrinolytic producer [64], serine protease isolated from *Fusarium* BLB were proposed to have fibrinolytic ability [65], 27.3 kDa fibrinolytic enzyme, CMase, is a metalloprotease produced from *Cordyceps militaris* [66,67], metalloprotease of 18.2 kDa obtained from a medicinal mushroom, *Schizophyllum commune,* was reported to have fibrin lysis activity [68], *Aspergillus oryzae* KSK-3-derived serine protease of 30 kDa exhibit thrombolytic potential [69], serine proteases from *Bionectria* sp. can be the potential treatment for thrombotic diseases [70], alkaline protease and metalloprotease obtained from *Aspergillus* strain KH 17 and *Aspergillus brasiliensis* AUMC 9735, respectively, were proposed to have robust fibrinolytic potential [71,72], and protease isolated from *Mucor subtilissimus* UCP 1262 via two different approaches—solid-state fermentation and aqueous two-phase system—were reported as a potential promising agent for the prevention and therapy against thrombosis [73].

#### 2.1.3. Algae

**Figure 5:**
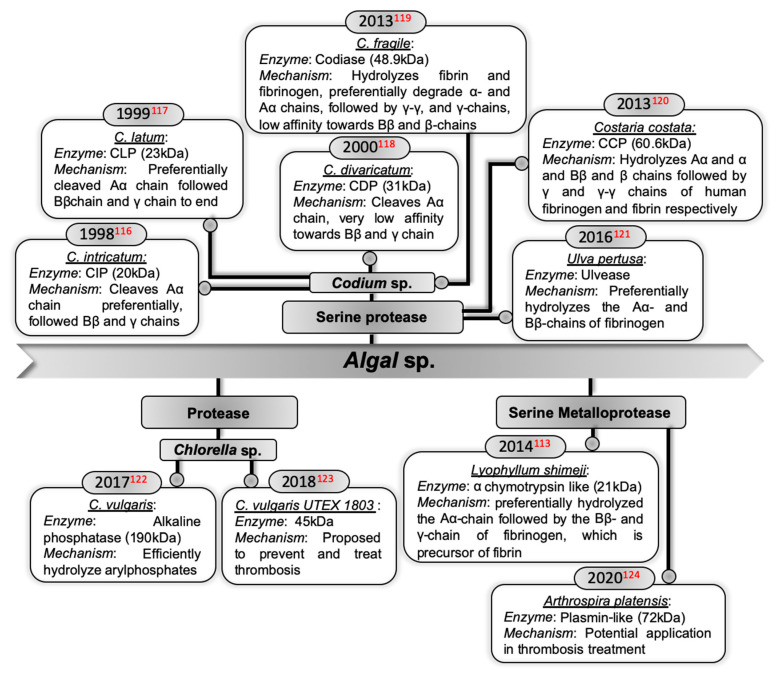
Fibrinolytic potential of algal-derived enzymes [113,116,117,118,119,120,121,122,123,124].

Algae are excellent sources of various biologically active agents with pleiotropic effects and are rich sources of food, feed, and energy. Few studies have evaluated the potential applications of algal-derived enzymes in fibrinolytic therapies (summarized in Figure 5), thus introducing a new era of bioprospecting of enzymes from algae for their role in thrombolytic activity. 

## 3. Microbial-Derived Drugs in Thrombolytic Therapy

### 3.1. Streptokinase

Streptokinase (SK) is the most commonly used first-generation thrombolytic agent. It is a 47 kDa protein and comprises 414 amino acid (AA) residues and exhibits its maximum activity at pH ~7.5 [125]. SK is isolated and derived from several strains of β-hemolytic *Streptococci* of Lancefield group A, C, and G [126,127]. The *Streptococcus equisimilis* GCS strain H46A and group C have been chiefly used for SK production [128]. SK comprises three structural domains: α, β, and γ at the 1–146, 147–290, and 291–414 AA positions catenated by two flexible coil regions. SK has a half-life of ~30 min [129,130]. The thrombolytic potential of SK was first acknowledged by Johnson and Tillett in 1952, where they observed that artificially induced intravascular clots within the ear vein of rabbits via sodium morrhuate were successfully dissolved after intravenous infusion of SK [131].

Moreover, the large-scale purification of human plasminogen achieved by Kline [132] allowed the direct application of plasmin as a clot buster. However, the major solicitude associated with direct use of plasmin or SK-mediated plasminogen activation may result in indiscriminate, systemic plasmin generation and substantial diminution of circulating PLG and α-2-antiplasmin [133,134], which can lead to a significant reduction in blood clotting ability, and consequently, a serious risk of hemorrhage. Hence, limiting the application of plasminogen activators as a therapeutic agent rather than circulating plasmin. Correspondingly, Tillette and Sherry reported accompanying adverse effects of SK, which causes pyogenic reactions with symptoms like arthralgia, occasionally nausea, and headache, etc., ergo, limiting the administration of multiple doses of SK [135]. Nevertheless, continued efforts have been made to achieve reduced pyrogenic reaction of SK by using various approaches such as structural and chemical modifications, liposomal entrapment or encapsulation, and domain fusion (Table 2); the recombinant SK (rSK) thus obtained possessed less antigenicity compared to wild-type SK [136,137]. Cloning the SK gene in non-pathogenic microbes permits the production of fortified rSK that eliminates the risk of infection by potentially pathogenic *Streptococci* [138].

#### 3.1.1. Action Mechanism

SK functions by hijacking the fibrinolysis cascade, where it stoichiometrically binds to plasminogen, inducing a conformational change in plasminogen to form an enzymatically active streptokinase–plasminogen (SK-PLG) complex [139,140]. SK-PLG complex is a highly specific protease that further cleaves other circulating PLG molecules and is capable of converting them to active serine protease, plasmin, that can degrade the fibrin clot via its specific lysine binding site [141,142]. There are several functional regions across SK domains, including: (i) α domain—the Asp41–His48 region between 1-59 AA residues regulates binding to PLG [140,143]. It additionally contains single residues (V19F, V35E, and S44K) for substitution that are crucial for SK-plasmin complex activation [142,144]; (ii) β domain—Lys 256, 257, and Val158–Arg219 region aid in PLG recognition, processing, and SK-plasmin complex formation [145]; and (iii) γ domain—a coiled region (Leu314-Ala342) that plays an essential role in stabilization of SK-micro plasmin complex for PLG activation. SK was the first Food and Drug Administration (FDA)-approved PLG activator for thrombosis therapy.

#### 3.1.2. A Potential Therapeutic Tool

Several clinical trials have been designed to determine the therapeutic potential and safety of SK. These trials concluded the increased survival rate in patients receiving SK during the early onset of myocardial infarction (AMI) [146,147,148]. Ruegsegger and colleagues were the first to prove SK-mediated intracoronary clot dissolution [149]. A subsequent study investigated the protective role of adjuvant intracoronary SK (ICSK) on late-phase infarct size and left ventricle volumes and functions in the setting of primary percutaneous coronary intervention (PCI). This study reported that immediate administration of a low dose of ICSK not only limits the long-term infarct size but also preserves left ventricle volumes and functions [150]. SK is, therefore, the drug of choice in thrombolytic therapy, especially in developing countries due to its reduced cost.

### 3.2. Staphylokinase (SAK)

SAK is a third-generation plasminogen activator obtained from *Staphylococcus aureus* GH38 which exerts its anti-thrombin activity by converting passive plasminogen to active plasmin. SAK is a 136 AA monomer of 15.5 kDa and comprises two equal-sized domains with flexible dumbbell shapes [95,158]. Structurally, it has been reported that AA at the 26th position (methionine-26) is useful for PLG activation by SAK. Notably, the functional activity is lost if this AA is replaced with arginine or valine, whereas little or no effect on functional activity was observed by replacing it with leucine or cysteine [159]. SAK was precipitated: (i) initially from the supernatant fluid of cultures at pH around 3.3 with 10 mM HCL; and also (ii) at 75% saturation of (NH_4_)_2_SO_4_ (ammonium sulfate) [160,161]. The half-life of SAK is ~6 min [162]. Lewis and Sweet evaluated the in vitro fibrinolytic properties of SAK [163,164]. Correspondingly, Lewis and Kanae observed in vivo thrombolytic activity of SAK in dogs [165].

#### 3.2.1. Action Mechanism

Fibrin degradation via SAK involves the following two steps: (i) formation of the SAK–PLG complex—this hydrolyzes a peptide bond between lysine 10 and 11 of SAK, which triggers peptide bond lysis between arginine 561 and valine 562 of PLG; (ii) resulting in the initial conversion of PLG to plasmin. SAK then binds to synthesized plasmin directly to catalyze the PLG conversion to plasmin [165]. α_2_-antiplasmin impedes the SAK-PLG or plasmin complex formation in the absence of fibrin [166,167]. However, the lysine binding domain of the complex is occupied in the presence of fibrin, preventing its inhibition by α_2_-antiplasmin [158,168]. This suggests the fibrin-specific nature of SAK, and that it can therefore be a potentially potent thrombolytic therapy [158].

#### 3.2.2. A Potential Therapeutic Tool

THR-174 is the optimized form of the SAK sequence variant produced by ThromboGenics NV, Iselin, NJ, USA documented to have augmented efficacy and safety profiles in the pre-clinical trials (2012. Official Website. http://www.thrombogenics.com—Retrieved online on 30 January 2021).

Furthermore, the 3-D structure of the protein facilitated the design of PEG (polyethylene glycol) attachment sites, prolonging plasma half-life and reducing antigenicity [169,170,171,172,173,174]. A recent study reported that N-terminal lipid modification of SAK fosters its activity, stability, and translocation across the blood–brain barrier (BBB), and therefore, holds great promise in treating diseases like stroke [175].

Studies have shown a key role of activated platelets in thrombosis, secondary clot formation, and blood vessel re-blocking. Following thrombolytic therapy, platelet aggregation promotes secondary clot formation. The coagulation cascade is activated by the ensuing clot lysis; in addition, a large amount of thrombin release elicits the platelets activation and aggregation. The activated platelets consequently inhibit fibrin lysis by tPA (tissue plasminogen activator) via the release of type I plasminogen activator inhibitor in the blood circulation, causing vessel re-blockage [176]. Interestingly, RGD (arginine, lysine, and aspartic acid tripeptide)–SAK complex bind to GPIIb/IIIa (glycoprotein membrane receptor) at the platelet surface, which averts the binding of fibrinogen to this receptor, thereby precludes the activated platelets accumulation [168]. Moreover, several thrombin inhibitors combined with recombinant SAK (rSAK) are known to decrease secondary clot formation [177,178,179,180,181,182].

### 3.3. Nattokinase (NK)

A serine protease derived from natto, a Japanese fermented food, was first extracted by Sumi and college [78] and was termed nattokinase. It is a 275-AA-long single polypeptide chain of 27.7 kDa. Nattokinase possesses the inherent ability to boost the endogenous mechanisms to degrade the blood clots and is achieved via following different ways: (i) oral administration—in vivo studies noted reduced euglobulin clot lysis time (ECLT), prolonged partial thromboplastin time (PATT), and averted platelet aggregation, therefore considered to be a direct-acting fibrinolytic enzyme [183,184]; (ii) intraduodenal administration—existing evidence support the transport of NK across the intestinal tract where it hydrolyzes the plasma fibrinogen [185]; (iii) efficacy—promotes fibrinolysis by degrading plasminogen activator inhibitor-1 (PAI-1) and aiding in plasmin formation by escalating the production of PLG activator [183,186]; and (iv) affinity—lower specificity and more affinity towards fibrinogen and cross-linked fibrin, respectively [187]. In summary, use of NK has several advantages, including efficacy in clot lysis and restoration of arterial blood flow in contrast with plasmin and elastase [188].

#### Clinical Trials

NK was shown to lower the plasma levels of fibrinogen FVII, and FVIII in two separate groups of patients undergoing dialysis and with cardiovascular risk factors without influencing blood lipids [189], enhanced fibrinolysis and anticoagulant activity [190], lower vWF levels [191], and is under Phase II trial to test its therapeutic ability against atherothrombosis [192].

### 3.4. Reteplase (Recombinant Plasminogen Activator, r-PA)

Reteplase or r-PA (39 kDa) is produced in *E. coli* K12 as insoluble inclusion bodies and is an unglycosylated single-chain deletion variant of tPA. Comprised of 1–3 and 176–527 AA of tPA, it therefore only contains the catalytic protease domains and kringle 2, and is deficient in the finger, kringle 1, and epidermal growth factor of t-PA, as signified by the deletion of the Val^4^-Glu [193,194]. Since the kringle 1 domain of r-PA is crucial for its rapid renal clearance, loss of kringle 1 domain aids in extending the half-life (~from 4 to 15 min) of r-PA [195,196]. Notably, the absence of the fibrin-binding finger domain in r-PA decreases (~5-fold) its binding affinity towards fibrin [194,197]. Interestingly, patients with acute myocardial infarction (AMI) administered with a double bolus regimen (10 + 10 M after 30 min) of r-PA showed robust thrombolysis activity [198,199]. Additionally, in The Global Use of Strategies to Open Occluded Coronary Arteries (GUSTO III) trial, hemorrhagic stroke frequency and mortality post-30 days were comparable in r-PA and alteplase [200]. r-PA is commercially available as Retavase^®^ (Centocor, Inc., Malvern, PA, USA) and Rapilysin^®^ (Roche) for AMI therapy.

## 4. Use of Waste Biomass/by-Products for Thrombolytic/Fibrinolytic Enzyme Production

There is a constant search for novel and safer fibrinolytic enzymes all over the world owing to the short life cycle and allergic reactions caused by the tPA and certain fibrinolytic agents [201]. The microbial fibrinolytic enzymes are quite economical, and have been studied in insects [202], marine organisms [203], and fermented foods [187,204]. Solid-state fermentation (SSF) is an efficient method for enzyme production and metabolite bioconversion. Agro-industrial waste, fishery waste, and wastewater can be used for the production of fibrinolytic enzymes. A wide variety of agro-wastes, such as groundnut husk [205], green gram husk [206], copra waste [207], deproteinized acid cheese whey and wheat bran [208], and cake of *Jatropha curcas* seed [201] have been effectively used for the production of enzymes. The solid substrate provides essential nutrients for both the growth of microbes and the production of enzymes. Various studies have shown that *B. subtilis* can produce a variety of fibrinolytic enzymes [209,210]. Vijayaraghavan and group [50] extracted fibrinolytic enzymes from *Bacillus halodurans* using agro-wastes. They observed wheat bran to be an efficient substrate for the production of fibrinolytic enzymes which could degrade fibrin clot thus behaving as an effective thrombolytic agent. Wu et al. [211] developed a cost-effective method to optimize the parameters of fermentation for the production of fibrinolytic enzymes by *Bacillus subtilis* WR350. Their results revealed that sucrose can be used as a low-cost substrate for the production of the fibrinolytic enzyme in a 100-L fermenter by *B. subtilis* WR350. Fish wastes also act as good substrates for the production of fibrinolytic enzymes due to their high nutrient content [212]. Biji et al. [201] produced fibrinolytic enzymes by *Bacillus cereus* IND5 using cow dung and cuttlefish waste in SSF. The purified enzyme obtained had a specific activity of 364.5 U/g proteins, the molecular weight of 47 kDa, stability at pH 8.0, high activity at 50 °C and was shown to possess fibrinolytic properties. Thus, the mixture of cuttlefish waste and cow dung had great applications as solid substrates for the production of fibrinolytic enzymes. Hence, use of waste biomass not only yields cost-effective and efficient fibrinolytic enzymes, but also reduces the chances of environmental pollution.

## 5. Conclusions and Future Perspectives 

Vascular occlusion remains a major cause of morbidity and mortality worldwide. Although numerous thrombolytic agents have been identified and characterized from diverse sources, promising scientific data available from in vitro and in vivo studies have failed to translate into clinical trials successfully. Therefore, continuous efforts are needed in the search for more efficacious, safer, and cost-effective thrombolytic drugs. Microbial-derived thrombolytic agents represent a step towards a potent approach in the prevention and treatment of vascular diseases such as CVDs, stroke, transient ischemic attack (TIA), PE, AAAs, venous thromboembolism (VTE), etc. Several thrombolytic enzymes have been reported to be isolated from microbial sources with therapeutic application in vascular diseases and have been shown to possess the following advantages over currently available treatment strategies: (i) extended plasma half-life, (ii) increased fibrin specificity, (iii) high therapeutic index, (iv) lower allergic response, and (v) reduced risk of bleeding complications. Therefore, they promise efficacious translational potential. Both PLG activators and plasmin-like enzymes have been reported to exhibit these advantages. Thus, thrombolytic and fibrinolytic enzymes isolated from microbial sources would spur novel therapeutic strategies for advancing the prospects of these microbial-derived enzyme complexes in the therapeutic armamentarium of drugs.

## Figures and Tables

**Table 1:** Overview of hemostasis [19,20,21].

Primary Hemostasis (Platelet Plug Formation)	Secondary Hemostasis (Coagulation Cascade to Form Fibrin Network)
It is a response to endothelial disruption	It is the phase of strengthening and stabilization of the soft platelet plug formed at the site of injury.
Injured endothelium exposes the procoagulant subendothelial matrix and promptly initiates primary homeostasis, which comprises of following four sequential but overlapping phases: Vasoconstriction - Mediated by endothelin-1 - Damaged endothelium exposes sub-endothelial collagen, von Willebrand factor (vWF), releases ATP, and inflammatory mediators Platelet adhesion - Gp Ib-IX receptor on platelets binds to vWF Platelet activation - Mediated via thrombin by 2 mechanisms: (1) Irreversible change in platelets shape from smooth discs to multi-pseudopodal plugs and (2) Secretion of cytoplasmic granules Platelet aggregation - Gp IIb/IIIa receptors adhere to vWF and fibrinogen	It initiates enzymatic activation of coagulation proteins to strengthen the soft platelet plug via stable fibrin clot formation, which involves several steps and can be divided into 3 pathways as follows: Extrinsic pathway - Initiates with binding of tissue factor (TF) and factor VII (FVII), activating FVII to factor VIIa (FVIIa), forming a *TF-FVIIa* complex - TF-FVIIa complex activates factor X (FX) to get FXa, initiating common pathway described below—(iii) Intrinsic pathway - Initiated with activation of FXIIa - FXIIa activates FXI to FXIa - FXIa activates FIX to FIXa - FIXa-factor VIII (FVIIIa) complex activates FX, initiating common pathway described below—(iii) Common pathway - Factor Xa + Factor Va + calcium on phospholipid surfaces (prothrombinase complex) = activation of activating prothrombin (aka Factor II) to thrombin. - Thrombin activates FXIIIa - FXIIIa crosslinks with fibrin forming stabilized clot
Limits bleeding instantly	Gradually fibrinolysis will dissolve the stable plug

**Table 2:** Summary of strategies used for SK modifications.

Modifications	Type	Examples	Mode of Action	References
Structural	Deletion	SK60-386 and SK143-386	Fibrin-specific activity, lower immunogenicity	[151]
	Substitution	Lys59 and Lys386 for glutamine	Increases half-life	[152]
Chemical	PEGylation	cysteine-specific thiol-mediated	Increases half-life and stability	[125]
	Acylation	Human plasminogen-bacterial streptokinase complex	Enhances specificity	[153]
Delivery system	Liposomal entrapment or encapsulation	PEG or chitosan nanoparticles, platelet directed liposomes	Increases half-life and stability, reduced immunogenicity, improved clot penetration properties	[154,155,156]
Domain fusion	Chimeric and conjugated protein	Fusion with epidermal growth factor 4, 5, and 6 domains of human thrombomodulin	Reduced risk of re-occlusion and hemorrhage	[157]

## Data Availability

Study did not report any data.
